# Oral Treatments With Probiotics and Live *Salmonella* Vaccine Induce Unique Changes in Gut Neurochemicals and Microbiome in Chickens

**DOI:** 10.3389/fmicb.2019.03064

**Published:** 2020-01-15

**Authors:** Graham A. J. Redweik, Karrie Daniels, Andrew J. Severin, Mark Lyte, Melha Mellata

**Affiliations:** ^1^Department of Food Science and Human Nutrition, Iowa State University, Ames, IA, United States; ^2^Interdepartmental Microbiology Graduate Program, Iowa State University, Ames, IA, United States; ^3^Department of Veterinary Microbiology and Preventative Medicine, Iowa State University, Ames, IA, United States; ^4^Genome Informatics Facility, Iowa State University, Ames, IA, United States

**Keywords:** probiotics, poultry, intestine, neurochemicals, *Akkermansia*, enterobacteriaceae, IgA

## Abstract

Cross-talk between the gut microbiota and neurochemicals affects health and well-being of animals. However, little is known about this interaction in chickens despite their importance in food production. Probiotics and live *Salmonella* vaccines are microbial products commonly given orally to layer pullets to improve health and ensure food safety. This study’s objective was to determine how these oral treatments, individually or in combination, would impact the gut environment of chickens. White Leghorn chicks were either non-treated (CON) or orally given probiotics (PRO), a recombinant attenuated *Salmonella* vaccine (RASV; VAX), or both (P+V). Birds were fed with probiotics daily beginning at 1-day-old and orally immunized with RASV at 4-days-old and boosted 2 weeks post-primary vaccination. At 5 weeks, ceca content, ceca tissues, and small intestinal scrapings (SISs) were collected from ten birds/group post-euthanasia for analyses. Catecholamine, but not serotonergic, metabolism was affected by treatments. Dopamine metabolism, indicated by L-DOPA and DOPAC levels, were increased in P+V birds versus CON and PRO birds. Based on 16S sequencing, beta diversity was more similar among vaccinated birds versus birds given probiotics, suggesting live *Salmonella* vaccination has a major selective pressure on microbial diversity. Abundances of *Akkermansia muciniphila* and Enterobacteriaceae positively correlated with levels of tyrosine and norepinephrine, respectively. Both enumeration and 16S sequencing, determined that PRO exhibited the greatest levels of Enterobacteriaceae in the ceca and feces, which was associated with greater IgA production against *E. coli* virulence factors as tested by ELISA. In summary, we demonstrate that using probiotics alone versus in combination with a live vaccine has major implications in catecholamine production and the microbiota of layer pullets. Additionally, unique correlations between changes in some neurochemicals and specific bacteria have been shown.

## Introduction

The gut microbiota directly regulates host activities through the brain-gut-enteric microbiota axis (Rhee et al., 2009). The ability of microbes to secrete and respond to neurochemicals, i.e., microbial endocrinology, has major implications on host health and behavior (Lyte, 2016), including poultry (Villageliú and Lyte, 2017). In poultry, probiotics are used by commercial producers to improve intestinal microbial balance, intestinal morphology, colonization resistance against pathogens, nutrient acquisition, animal performance, and immune responses in chickens (Fuller, 1989; Patterson and Burkholder, 2003; Lutful Kabir, 2009). In commercial poultry practices, lactic acid bacteria (e.g., *Lactobacillus acidophilus*, *Pediococcus* spp.), yeast (e.g., *Saccharomyces* spp.), and spore-formers (e.g., *Bacillus subtilis*) are probiotics commonly given to animals, typically as polymicrobial mixtures (FAO, 2016). Additionally, probiotics can serve as delivery vehicles for neuroactive compounds (Lyte, 2011), suggesting probiotics may change the dynamics of neurochemical production in the chicken intestine.

Live *Salmonella* vaccines are commonly used to reduce colonization of broad-host *Salmonella enterica* serovars in poultry (Hassan and Curtiss, 1997), the primary vehicle of human salmonellosis (Batz et al., 2012). These vaccines successfully reduce *Salmonella* ceca colonization (Muniz et al., 2017) and environmental contamination (Dórea et al., 2010). Given that *S. enterica* serovar Typhimurium virulence is stimulated by norepinephrine and epinephrine (Karavolos et al., 2008; Pullinger et al., 2010), *S. Typhimurium*-derived vaccines could also respond to neurochemicals. Microbial endocrinology research is virtually absent in chickens (Villageliú and Lyte, 2017), and no study has investigated the impact of oral vaccination on neurochemical synthesis in the gut of any animal model. Recently, we found that layer pullets orally treated with a commercial probiotic mix and live attenuated *Salmonella* vaccine χ9373 improved resistance to *E. coli* air sac challenge and *Salmonella* gut colonization (Redweik et al., 2019). However, the effects of these oral treatments on the gut microbiota and neurochemical production are unknown. In this study, we establish a foundation for microbial endocrinology in the chicken gut by evaluating (i) neurochemical production and (ii) microbiome in the ceca of chickens given probiotics, a recombinant attenuated *Salmonella* vaccine (RASV), or both. We hypothesize that each treatment group will have a unique neurochemical and microbiome profiles.

## Materials and Methods

### Ethics Statement and General Conditions

This project was approved by Iowa State University Institutional Animal Care and Use Committee, log #1-16-8159-G. Animals were placed in open-floor pens and given enrichments (i.e., string-hung CDs, free range of movement) during the course of the experiment. Euthanasia techniques (CO_2_ asphyxiation followed by thoracotomy) followed the American Veterinary Medical Association Guidelines (2013).

### *In vivo* Experiments

**FIGURE 1:**
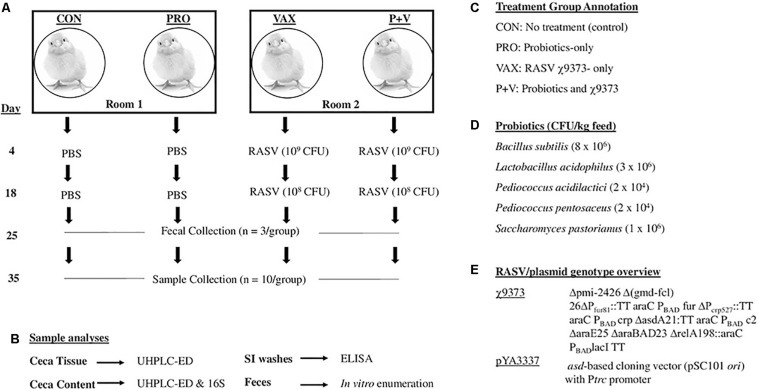
Overview of present study. Four groups of birds were evenly split into two rooms, one group given probiotics per room. At 4 and 18 days, birds received recombinant attenuated *Salmonella* vaccine χ9373 (RASV) or PBS as a control. Samples were collected at days 25 and 35 **(A)**, which were then used for subsequent analyses **(B)**. Summary of abbreviations used in this study for treatment groups **(C)**. Probiotic CFUs in mix **(D)**. Genotypic details of the RASV **(E)**.

1-day-old male and female specific pathogen-free layer chickens (White Leghorns; VALO, Adel, IA, United States) were randomly placed into four pens (*n* = 10 birds/pen), evenly split between two rooms to separate unvaccinated and vaccinated chickens, respectively (Figure 1A). Chickens were given *ad libitum* access to feed and water. One pen per room received a commercial probiotic supplement (Gro-2-Max, BioNatural America Institute), containing *Bacillus subtilis*, *Lactobacillus acidophilus*, *Pediococcus acidilactici*, *Pediococcus pentosaceus*, and *Saccharomyces pastorianus*, as verified phenotypically and by PCR, thoroughly mixed with feed (2.5 g dry probiotic mix to 2.3 kg feed; PRO and P+V, Figures 1C,D). Fresh feed was evenly weighed and replaced in each pen every 2 days. Bedding was not replaced during the duration of the experiment.

### Vaccine Preparation and Immunization

Recombinant attenuated *Salmonella* vaccine χ9373 (Figure 1E) was derived from the virulent *S. Typhimurium* strain UK-1 (χ3761), using molecular strategies that enhance safety and immunogenicity (Curtiss et al., 2010). This strain was previously shown to effectively colonize chickens *in vivo* (Pei et al., 2014). The day prior to vaccination, χ9373 was cultured in Lysogeny Brothe (LB) broth (0.1% glucose, 0.02% mannose, 0.05% arabinose) overnight at 37°C. The next day, χ9373 was grown shaking in the same media until OD_600_ reached ∼0.8, and the inoculum was centrifuged for 20 min at 4,000 × *g* at room temperature. The pellet was then resuspended, serially diluted in PBS, and plated on MacConkey agar to confirm bacterial concentrations.

At 4-days-old, feed and water were removed from pens of all birds 4–6 h prior to vaccination. Chickens in the vaccine groups (i.e., VAX and P+V) were orally immunized via micropipette with 20 μl of 10^9^ colony-forming units (CFU) χ9373. Two weeks post-primary vaccination, the same chickens were orally immunized with a 20 μl χ9373 boost (10^8^CFU). Non-vaccinated birds (i.e., CON and PRO) received 20 μl PBS as a control. Feed and water were returned to pens 30 min post-immunization. Enumeration of χ9373 in feces (*n* = 5) has shown no differences in colonization in VAX and P+V chickens (Supplementary Figure 1).

### Sample Collection

Sample collection and corresponding analyses are summarized in Figures 1A,B, respectively. At 4 weeks of age, three birds per group were randomly selected and placed into sterile containers to collect feces. Fecal matter was then resuspended in PBS, serially diluted, and plated onto MacConkey agar for Enterobacteriaceae enumeration. At 5 weeks of age, birds were humanely euthanized via CO_2_ asphyxiation. To collect small intestinal scrapings (SISs; *n* = 10 per group), a 10-cm segment aligning Meckel’s diverticulum in the center was longitudinally cut to expose the lumen. After removing excess luminal contents, the epithelial layer was gently scraped and then washed with 1 ml PBS to collect mucus into 50 ml conicals (one conical/bird) filled with 10 ml PBS. Conicals were then centrifuged at 5,000 × *g* for 20 min at 4°C. Then, 1 ml supernatant was added to 30 μl storage mixture (1% sodium azide, 5% BSA, 50 mM phenylmethane sulfonyl fluoride) and stored in −80°C.

To collect ceca samples (*n* = 10 per group), contents were squeezed from both ceca into respective cryogenic tubes (Nalgene System 100^TM^, Thermo Fisher Scientific) and immediately placed on dry ice. The remaining ceca tissue (*n* = 10 per group) was briefly washed with PBS and flash-frozen in liquid nitrogen, and the tissues were transferred to cryogenic tubes and placed on dry ice. Tubes were then moved into −80°C for long-term storage.

### Ultra-High Pressure Liquid Chromatography

Ceca tissue, content, and SISs were pre-treated with 0.2 M perchloric acid (1:10 sample-acid ratio), and homogenized via Omni Bead Ruptor tubes. After centrifugation, supernatant liquid was transferred to 2–3 kDa spin filter and centrifuged again at 2,950 × *g* at 4°C. Flow-through was then analyzed via ultra-high-performance liquid chromatography with electrochemical detection (UHPLC-ED) using the 99 Dionex UltiMate 3000 with MD-TM Mobile Phase Solution as sample diluent (Fisher Scientific) as performed previously (Villageliú et al., 2018).

### DNA Isolation and Microbiome 16S rRNA Sequencing

Total DNA was isolated from 0.25 g of ceca contents using the DNeasy PowerSoil Kit (Qiagen): Extracted DNAs were assessed for quality using a NanoDrop 2000 spectrophotometer 260–280 nm ratios. Concentrations were determined using a Qubit fluorometer with the double-stranded DNA broad range kit (Thermo Fisher Scientific), adjusted to 50 ng/μl in nuclease-free water, and shipped on dry ice to Argonne National Laboratory in Lemont, IL, United States. All 40 ceca samples were used for sequencing. DNAs were used for library preparation using the MiSeq and HiSeq2500 kit (Illumina) following all manufacturer’s instructions with 151 × 151 paired-end MiSeq sequencing (Illumina). For 16S analysis, QIIME2 (version 2019.10) was used to analyze the 16S data between all sequenced groups. However, due to lane effect (further details provided in our GitHub repository at^1^), all CON and four PRO samples were removed. Thereafter, QIIME2 analysis was used with the remaining samples to compare treatment groups PRO, *n* = 6; VAX, *n* = 10; and P+V, *n* = 10). Sequences were demultiplexed using the QIIME2 demux emp-paired function and denoised using the QIIME2 plugin DADA2. The number of good quality reads for taxonomic assignment ranged from 27,272 to 60,368 reads. GreenGenes database (version 13.8) at the 99% operational taxonomic units (OTUs) for the region (515F/806R) was used to classify each of the reads using QIIME2’s feature-classifier function. Alpha and beta diversity analyses were calculated using QIIME2’s built in functions. Gneiss plugin was used to explore taxonomic balances and taxonomic group differences between treatment groups. The ols-regression summary indicated the model used (Treatment+Unknown) was a good fit for the data with small residuals. A large unknown factor was noted to account for 40% of the variation but this variation was orthogonal to the variation that can discriminate between treatment groups and can be safely ignored. For a more thorough description of our step-by-step methods, please refer to the GitHub repository at https://github.com/ISUgenomics/MelhaMellata. The 16S dataset is available in the NCBI Sequence Read Archive (SRA) repository with accession BioProject ID SUB5641933.

### IgA Titers Measured by ELISA

Ninety-six-well plates were coated with 2.0 μg/ml of lipopolysaccharide (LPS, *Salmonella enterica* serovar Typhimurium, Sigma), salmochelin receptor (IroN), aerobactin receptor (IutA), or 0.25 μg/ml unlabeled chicken IgA (i.e., total IgA; H+L, Thermo Fisher Scientific) overnight at 4°C. LPS is common in gram-negative bacteria, and IroN and IutA are virulence factors involved in iron acquisition. Recombinant IroN and IutA proteins were purified from culture of *E. coli* BL21 containing the pET-101/D-TOPO vectors (Invitrogen) carrying *iroN* or *iutA* genes as previously described (Mellata et al., 2016). SISs were diluted 1:1 in SEA blocking buffer (Thermo Fisher Scientific), serially diluted 1:2, and incubated for 1 h at room temperature. Goat-anti-chicken-IgA-AP (H+L, Thermo Fisher Scientific) was added, followed by PNPP substrate (Thermo Fisher Scientific), and absorbance was measured at 405 nm. To measure antibody titer, the reciprocal of the highest dilution values doubling the control value (i.e., CON birds) were considered positive. ELISAs were done in duplicate per individual bird and independently replicated twice.

### Statistical Analysis

Statistical analyses were performed using GraphPad Prism software 6.0 for non-linear regression analyses. One-way ANOVA followed by Tukey’s test for multiple means comparisons was used to compare differences between all groups for each experiment. *P*-values < 0.05 were considered significant. For correlational analyses, R software was used to run linear regression models between log-transformed 16S reads of microbial taxa (normalization step) and neurochemical metabolite concentrations. Log transformations were done in Excel, and data used for linear regression analyses are included in Supplementary Table 1. Improved normalization of 16S abundances via log transformation can be seen in Supplementary Figures 7,8.

## Results

### Catecholamine, but Not Serotonin Metabolism, Was Altered in Treatment-Specific Manner

**FIGURE 2:**
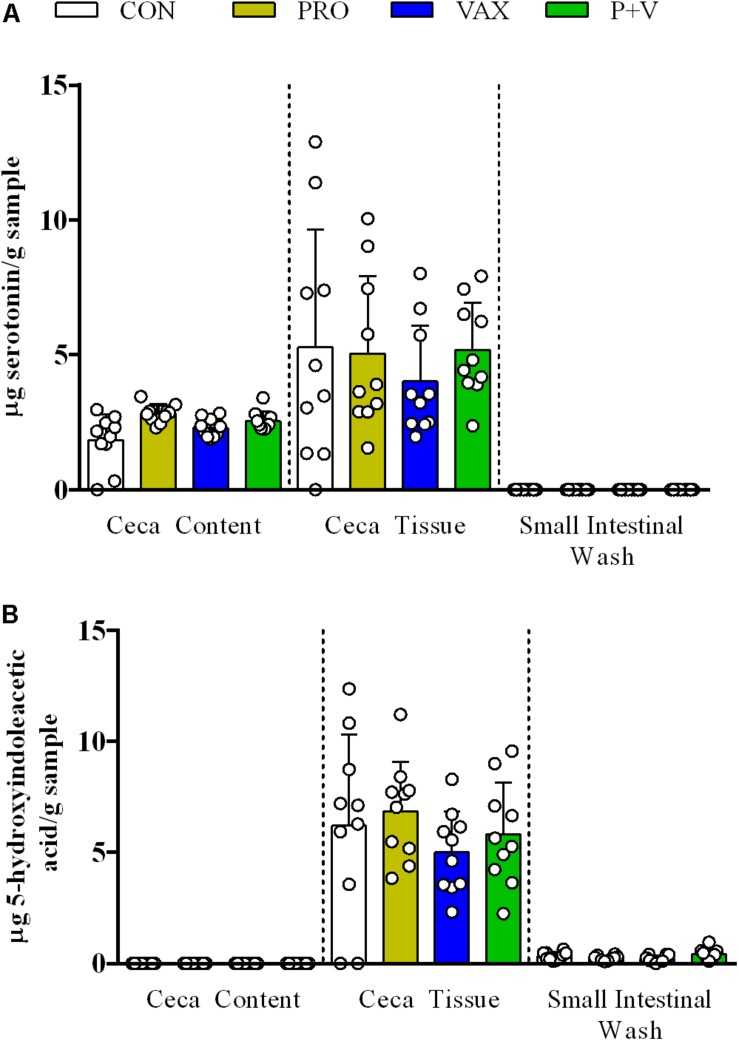
Detection of serotoninergic metabolites **(A)** serotonin and **(B)** 5-HIAA acid in ceca content, ceca tissue, and small intestinal scrapings. Each dot represents an individual animal, bars represent mean ± standard deviation. White, CON; yellow, PRO; blue, VAX; green, P+V.

**FIGURE 3:**
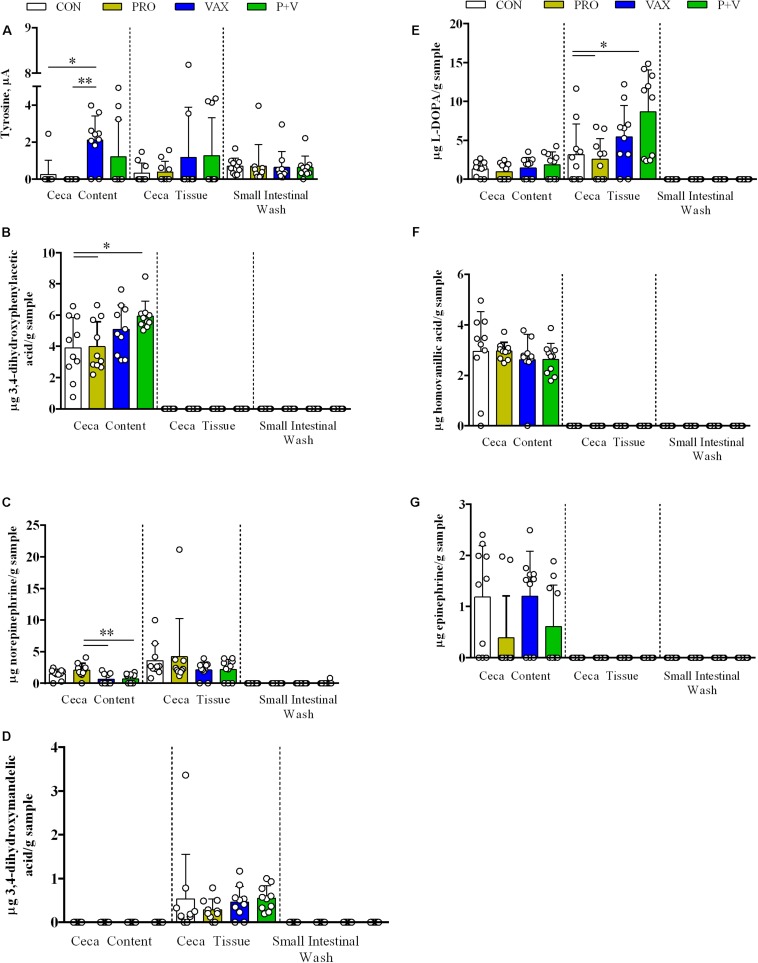
Detection of catecholamine metabolites **(A)** tyrosine, **(B)** DOPAC, **(C)** norepinephrine, **(D)** DHMA, **(E)** L-DOPA, **(F)** HVA, and **(G)** epinephrine in ceca content, ceca tissue, and small intestinal scrapings. Each dot represents an individual animal, bars represent mean ± standard deviation. ^∗^*P* < 0.05; ^∗∗^*P* < 0.01. White, CON; yellow, PRO; blue, VAX; green, P+V.

Total neurochemical metabolites detected in any tissue type were split by metabolic pathway, i.e., serotonergic (Figure 2) and catecholaminergic (Figure 3). In all treatment groups, serotonin was detected in both the ceca content and tissue but not in SISs (Figure 2A). Its breakdown product, 5-hydroxyindoleacetic (5-HIAA), was similarly detected in the ceca tissue (Figure 2B) but not in the content. Additionally, 5-HIAA was detectable in SISs. Treatment did not significantly change production of either metabolite in any sample.

In Figure 3, catecholamines were consistently detected in ceca content and tissue, though only tyrosine levels reached a detectable threshold in SISs (Figure 3A). In general, treatments modified metabolite levels in a case-by-case basis. In Figure 3A, the ceca content of VAX birds exhibited increased tyrosine levels compared to CON (*P* < 0.05) and PRO (*P* < 0.01). Ceca content from P+V birds had the highest levels of 3,4-dihydroxyphenylacetic acid (DOPAC) compared to both CON and PRO birds (*P* < 0.05; Figure 3B). PRO birds exhibited increased norepinephrine levels in ceca content versus VAX and P+V birds (Figure 3C; *P* < 0.01). L-3,4-dihydroxyphenylalanine (L-DOPA) levels were dramatically increased in P+V tissue compared to CON (*P* < 0.05) and Probiotics (*P* < 0.05; Figure 3E). No differences were found for levels of 3,4-dihydroxymandelic acid (DHMA, Figure 3D), homovanillic acid (HVA, Figure 3F), and epinephrine (Figure 3G) in any sample tested between treatment groups.

### Ceca Microbiome Diversity Changed by Treatment

Microbiome 16S sequencing and analyses were originally performed with ten samples from each group (CON, PRO, VAX, and P+V). However, after detecting a lane effect that affected our 16S analysis, we corrected this effect by focusing on analyzing those sequenced on a single lane only, which includes the three treatment groups (PRO, VAX, and P+V),. Despite this limitation, this strategy still allows ceca microbiome characterization of chickens given P+V versus mono-treated animals, PRO or VAX.

**FIGURE 4:**
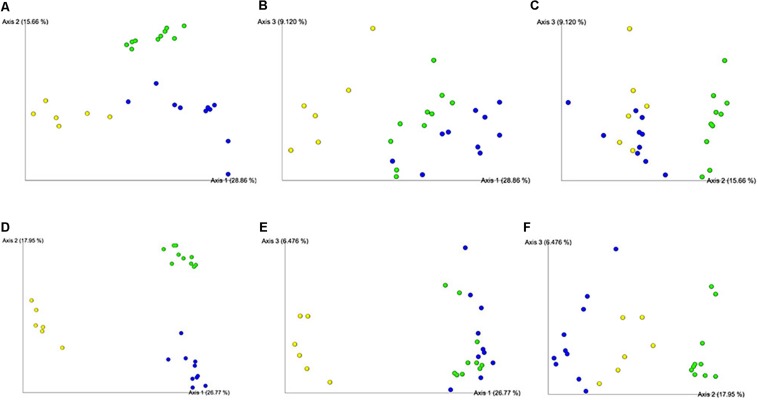
Beta diversity plots of ceca microbiome from individual birds via Bray–Curtis **(A-C)** and Jaccard **(D-F)** PCoA plots. Each sphere represents an individual bird, colored per respective group. Figures were generated by QIIME2 software. Yellow, PRO; blue, VAX; green, P+V.

Using multiple means comparisons via one-way ANOVA, microbial richness (i.e., Faith’s PD) was not statistically different between treatment groups (Supplementary Figure 2A). However, evenness was significantly greater in P+V versus VAX group (*P* < 0.05; Supplementary Figure 2B), but no significant difference between P+V and PRO was observed. Using a Bray–Curtis PCoA and a Jaccard Emporer plot to display quantitative (Figures 4A–C) and qualitative (Figures 4D–F) differences for community dissimilarity, respectively. These data show that ceca microbiomes of VAX and P+V birds clustered similarly along Axes 1 and 3 of the respective diversity plots. Overall, individuals tended to cluster based on treatment group.

### Specific Microbial Abundances Are Influenced by Live Prophylactics

**FIGURE 5:**
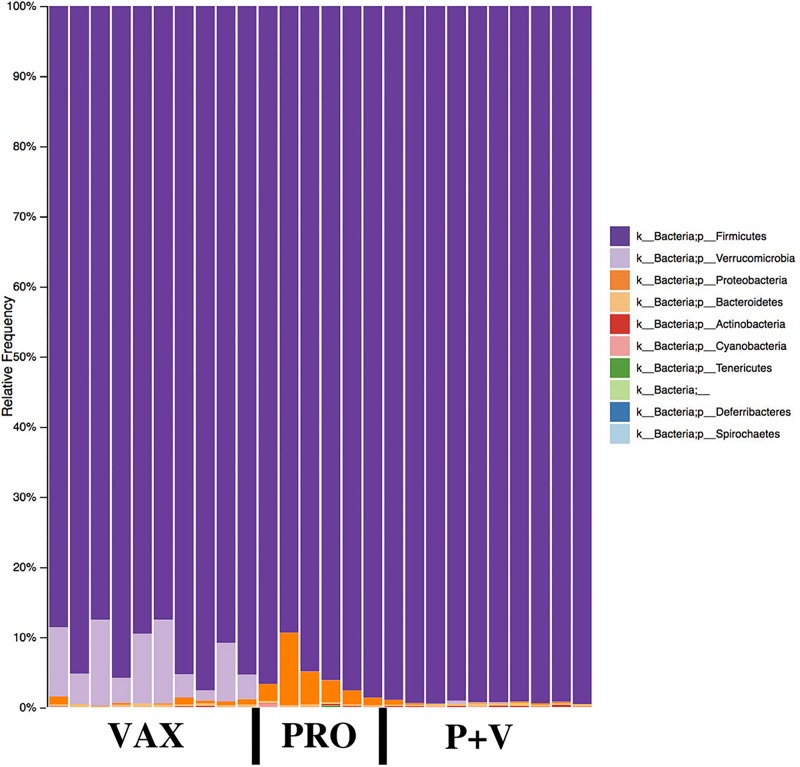
Microbial phyla relative frequencies in the chicken ceca by treatment group. Frequencies were generated by QIIME2 software. VAX, vaccine-only. PRO, probiotics only. P+V, vaccine and probiotics combination.

At the phylum level, Firmicutes were the most abundant phylum in all groups (Figure 5). Proteobacteria and Verrucomicrobia were specifically increased in PRO (all groups, *P* < 0.001) and VAX (all groups, *P* < 0.001), respectively (Figure 5). At the class level, Clostridia were slightly lower in abundance in P+V versus PRO (*P* < 0.05; Supplementary Figure 3). Similar to the pattern of Proteobacteria abundances, PRO exhibited the greatest abundances of Gammaproteobacteria (all groups, *P* < 0.001; Supplementary Figure 3). At the family level, Lachnospiraceae levels were highest in PRO versus P+V birds (Supplementary Figure 4A, *P* < 0.01), and Peptostreptococcaceae were elevated in P+V versus VAX birds (Supplementary Figure 4B, *P* < 0.01). Additionally, Mogibacteriaceae were reduced in vaccinated birds versus PRO (Supplementary Figure 4C, *P* < 0.05).

**FIGURE 6:**
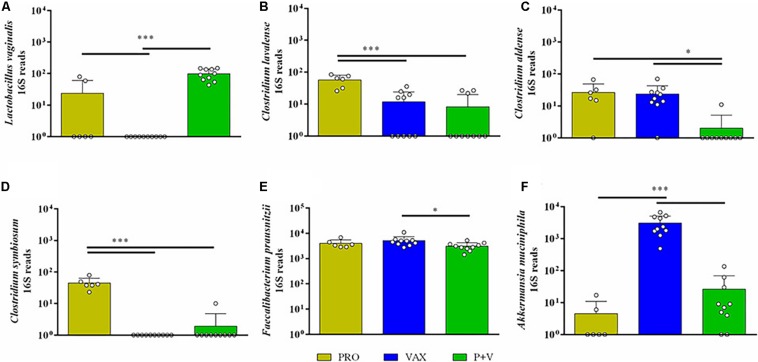
Bacterial abundances of bacterial species influenced by treatment group. 16S reads for **(A)** *Lactobacillus vaginalis*, **(B)** *Clostridium lavalense*, **(C)** *Clostridium aldense*, **(D)** *Clostridium symbiosum*, **(E)** *Faecalibacterium prausnitzii*, and **(F)** *Akkermansia muciniphila* were generated by QIIME2 software, and figures were developed on GraphPad. Yellow, PRO; blue, VAX; green, P+V. ^∗^*P* < 0.05; ^∗∗∗^*P* < 0.001.

At the genus level, birds from both vaccinated groups (i.e., VAX and P+V) had reduced levels of *Enterococcus*, *Weisella*, *Anaerofustis*, *Clostridium*, and *Coprabacillus* versus PRO (Supplementary Figures 5A–E; *P* < 0.001). Conversely, the Erysipelotrichaceae taxon *Cc-115* levels were elevated in P+V birds versus PRO (Supplementary Figure 5F; *P* < 0.05). Looking at the species level, *Lactobacillus vaginalis* levels were distinctly lower in VAX birds (CON and P+V, Figure 6A; *P* < 0.001). *Clostridium* species were impacted by treatment, as *C. lavalense* and *C. symbiosum* were decreased in vaccinated birds versus PRO (Figures 6B,D, *P* < 0.001). However, *C. aldense* was decreased in P+V birds alone versus PRO and VAX (Figure 6C, *P* < 0.05). *Faecalibacterium prausnitzii* levels were decreased in P+V versus VAX (Figure 6E, *P* < 0.05). Lastly, *Akkermansia muciniphila*, a member of the Verrucomicrobia phylum, were starkly elevated in VAX birds versus PRO and P+V (Figure 6F, *P* < 0.001).

Additionally, a Gneiss heatmap was used to construct microbial balance trees between treatment groups (Supplementary Figure 6 and Supplementary Table 2). Given estimating abundance levels inherently has its own limitations given lack of ability to absolutely quantify bacteria via 16S analyses (Morton et al., 2017), we used these data to support abundance shifts of specific taxa as well as identifying other taxa which could facilitate these shifts (hence their placement in these nodes via similar changes in balance). Full microbial balance data are provided in our GitHub repository (see text footnote 1). Looking at OTUs, PRO birds were distinguished from VAX and P+V birds, and Enterobacteriaceae, *Weisella*, *Anaerofustis*, *Coprabacillus* were among the 1,384 total OTUs specific to PRO birds within respective balances, supporting our previous findings. Furthermore, looking at the taxa level 463 OTUs were unique to VAX birds, including *Akkermansia muciniphila*, providing support that this taxon is specific to VAX birds. Lastly, 69 total OTUs were unique to P+V birds, including *Cc-115* (Supplementary Table 1).

### Enterobacteriaceae Antigen-Specific IgA Positively Associated With Enterobacteriaceae Levels

**FIGURE 7:**
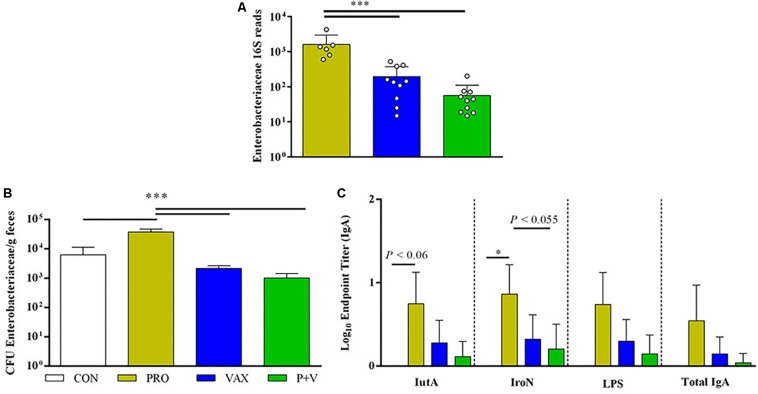
Associations between Enterobacteriaceae levels in ceca, feces and antigen-specific IgA production. **(A)** Enterobacteriaceae 16S reads generated by QIIME2 (*n* = 10 per group). **(B)** Enterobacteriaceae enumerated on MacConkey from feces (*n* = 3 per group, experimentally duplicated). **(C)** Antigen-specific and total IgA levels in small intestinal scrapings (IutA, aerobactin; IroN, salmochelin; LPS, lipopolysaccharide; experimentally duplicated). White, CON; yellow, PRO; blue, VAX; green, P+V. ^∗^*P* < 0.05; ^∗∗^*P* < 0.01; ^∗∗∗^*P* < 0.001.

In line with Enterobacteriaceae 16S abundance (Figure 7A), PRO birds had highest abundance of fecal Enterobacteriaceae determined by plating (Figure 7B; all groups, *P* < 0.001). Using ELISA to assess IgA in SISs, PRO scrapings yielded greatest Enterobacteriaceae-specific IgA levels compared to other groups (Figure 7C).

### Certain Bacteria Positively Correlated With Specific Neurochemical Metabolites

**FIGURE 8:**
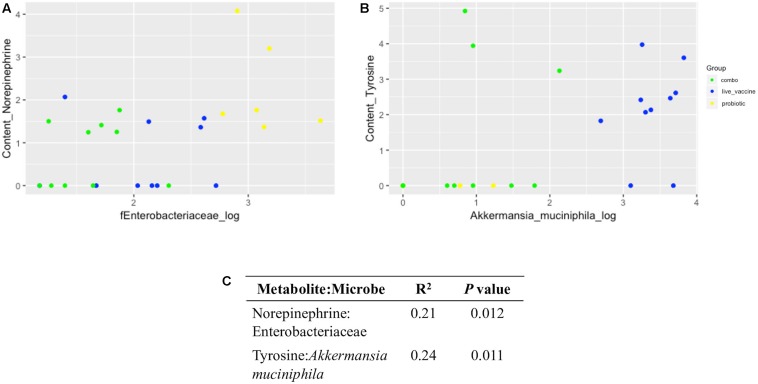
Linear regression plots and output between neurochemical metabolites and microbial taxa in ceca content. Data were generated via R software. **(A)** Norepinephrine:Enterobacteriaceae; **(B)** tyrosine: *Akkermansia muciniphila*; **(C)**, coefficient of determination (*R*^2^) and *P* value for each correlation. Yellow, PRO; blue, VAX; green, P+V.

Using a log transformation, there was a clear improvement in normalization of 16S data (Supplementary Figures 7, 8), improving conditions for performing a linear regression model. Significant, though weak, positive correlations were found between norepinephrine and Enterobacteriaceae (*R*^2^ = 0.21, *P* = 0.012; Figure 8A) and tyrosine and *A. muciniphila* (*R*^2^ = 0.24, *P* = 0.011 Figure 8B).

## Discussion

### Chicken Ceca Is a Major Site for Neurochemical Metabolism, Although Only Catecholamines Were Affected by Treatments

The impact of the gut microbiota on mammalian host behavior and emotions through neurochemical intermediates has been well-characterized (Rhee et al., 2009; Cryan and Dinan, 2012; Lyte, 2016). For example, spore-forming bacteria elevate tryptophan hydroxylase activity by colonic enterochromaffin cells, producing serotonin for local signaling or circulatory transportation (Wikoff et al., 2009; Yano et al., 2015). Some probiotics can produce neurochemicals like GABA, which bind to cognate receptors on the intrinsic primary afferent neurons innervating the intestinal villi (Forsythe and Kunze, 2013) or the epithelium itself (Cryan and Dinan, 2012). To the authors’ best knowledge, this is the first study to map neurochemicals in the chicken gut and correlate them to specific members of the chicken gut microbiota. The chicken cecum is the primary site for microbial fermentation in the gut (Svihus and Classen, 2013). Thus, the cecum content serves as a potential reservoir of neurochemical metabolites derived from microbial synthesis, although the host secretes some neuroactive chemicals into the lumen (Lyte, 2014).

In the avian brain, serotonin signaling plays a major role in aggression (Dennis et al., 2008) and exploratory behaviors (Dennis et al., 2013). In this study, we found no differences in serotonin metabolism in the chicken ceca between any treatment group. Notably, serotonin (and not 5-HIAA) was consistently detected in the ceca content, which suggests serotonin is selectively secreted into the intestinal lumen. This likely occurs via apical secretion by enterochromaffin cells (Fujimiya et al., 1997). This release can occur via direct control from the microbiota (Yano et al., 2015) as well as a means for the host to regulate these commensal microbes (Kwon et al., 2019). Although we did not find any differences in ceca serotonin metabolism between treatment groups, there is evidence live prophylactics can have an influence. In the zebrafish brain, supplementation of *Lactobacillus rhamnosus* IMC 501 modulated transcription of enzymes involved in serotonin production (Borrelli et al., 2016). Future studies could focus on the avian brain to further investigate the extraintestinal impacts of probiotics and live vaccines.

In this study, treatments uniquely affected levels of catecholamines in both ceca tissue and content. L-DOPA, the precursor to dopamine, was detected at the highest levels in ceca tissue in P+V birds. Furthermore, L-DOPA levels were associated with 3,4-dihydroxyphenylacetic acid (DOPAC), the waste metabolite of dopamine. Thus, it appears the combination of probiotics and RASV increased L-DOPA synthesis in the ceca, which resulted in excretion of dopaminergic waste metabolites primarily in the form of DOPAC. Notably, overall levels of homovanillic acid (HVA) and DOPAC combined were much greater than the sum of norepinephrine and epinephrine. This suggests intestinal dopamine in birds may be predominately degraded into waste metabolites rather than being utilized for catecholamine synthesis, aligning with what is observed in mammals (Kopin, 1985; Lambert et al., 1991; Eisenhofer et al., 1997). Increased L-DOPA in the chicken ceca tissue may be an indication of an increased abundance of non-neural cellular populations in the lamina propria (Eisenhofer et al., 1997) like regulatory T cells (Tregs), which express high levels of tyrosine hydroxylase (Cosentino et al., 2007) and play a crucial role in maintaining gut homeostasis (Barnes and Powrie, 2009). Current studies are underway to determine whether changes Treg abundances or other functions like gut motility (Li et al., 2006) are related to L-DOPA concentrations in the ceca.

### RASV Causes Major Rift in Gut Microbiome Diversity

The RASV χ9373 contains a number of genetic modifications, including a *pmi* deletion (Pei et al., 2014). This particular gene encodes 6-phosphomannose isomerase, which when missing ablates lipopolysaccharide (LPS) synthesis in the absence of mannose, resulting in increased complement and macrophage-mediated lysis of the bacterium (Curtiss et al., 2010). Other live *Salmonella* vaccines contain similar genetic attenuations to reduce virulence *in vivo* (Curtiss et al., 2010).

In young chicks, wild-type *Salmonella* infection causes major rifts in the ceca microbiome (Liu et al., 2018). Despite its attenuation, the RASV given to the chicks in this study drastically reduced beta diversity in treated groups, particularly decreasing the abundances of short chain fatty acid (SCFA)-producing fermenters like *Clostridium* [reviewed in Lopetuso et al. (2013)] and *Weisella* (Patel et al., 2013). These reductions may have negative consequences on chicken health, as SCFAs have numerous benefits for the host (Koh et al., 2016; Wu et al., 2018). Since the RASV was given at 4 days old, it likely triggered an inflammatory response, which altered the gut microbiome. Additionally, in this study, treating chickens with probiotics prior to RASV immunization, could have allowed birds to acquire a gut microbiota, which could have improved vaccine response (Ciabattini et al., 2019) and disease resistance (Redweik et al., 2019). However, future studies could test different *Salmonella* vaccines (genetic attenuations, serotype, etc.) as well as their effects at different time points.

### Certain Gut Bacteria Were Correlated With Neurochemical Metabolites

A positive correlation was detected between tyrosine and *A. muciniphila* levels, as both were higher in birds given the RASV only. *A. muciniphila* is a mucus-degrading bacterial taxon, which has garnered much interest due to its implicated health benefits (Naito et al., 2018). Mucin, produced by goblet cells in the intestinal tract and the major component of mucus, is a glycoprotein composed of several amino acids including tyrosine (Smirnov et al., 2004). Thus, it is possible the increase in *A. muciniphila* in the chicken ceca results in greater mucin degradation and, subsequently, tyrosine levels, which might directly affect bacterial abundances in the gut via its use as substrate (Mardinoglu et al., 2015).

Although oral live vaccines can improve mucosal immune responses (Kim and Jang, 2017), it has not been reported these live vaccines increase mucus production as well. Thus, given the lack of improved IgA production upon RASV immunization in this study, it is possible this live vaccine could have stimulated an increase in mucus production, improving intestinal barrier integrity. LPS (Smirnova et al., 2003) and wild-type *S. Typhimurium* (Zarepour et al., 2013) increase mucin production directly, suggesting this RASV could have induced a similar response. Interestingly, the addition of probiotics to the RASV ablated this effect. Future studies will seek to directly measure mucus thickness in vaccinated birds and how probiotics may interfere with mucin biosynthesis.

Our study has confirmed the positive relationship between norepinephrine and Enterobacteriaceae. Norepinephrine has been demonstrated to increase the growth of Enterobacteriaceae pathogens (Freestone et al., 2007a, b) through quorum sensing (Clarke et al., 2006). In this study, PRO birds exhibited the greatest levels of both norepinephrine and Enterobacteriaceae 16S reads in the ceca content, and this was supported by corresponding levels of Enterobacteriaceae fecal shedding. This observation seemingly contradicts the reputation of probiotics to inhibit GI pathogen colonization in the host (Patterson and Burkholder, 2003; Lutful Kabir, 2009). However, this response to norepinephrine is not limited to pathogenic Enterobacteriaceae, as non-pathogenic *E. coli* also respond to norepinephrine (Pasupuleti et al., 2014).

It is unlikely our probiotics are increasing norepinephrine production by the enteric nervous system, as there were not corresponding increases in metabolites, which would precede norepinephrine biosynthesis (i.e., L-DOPA) in the PRO group. Norepinephrine is commonly deactivated by the host during excretion via glucuronide conjugation. Notably, *E. coli* use beta-glucuronidase to deconjugate this form of norepinephrine in the gut (Asano et al., 2012). Thus, it is likely the probiotic mix increased commensal Enterobacteriaceae abundance, and this increase resulted in greater conversion of norepinephrine to its free form in the ceca. Whether increased availability of norepinephrine has an impact on the virulence of these Enterobacteriaceae (given its role as a quorum sensing ligand) remains to be investigated.

Furthermore, *E. coli* respond to norepinephrine via sensor kinase QseC, which upon activation increase transcription of *tynA* and *feaB*, whose protein products convert norepinephrine to DHMA (Pasupuleti et al., 2014). Although these authors proposed gut *E. coli* or other resident microbes convert norepinephrine to DHMA, in the present study we did not find corresponding levels of DHMA in the ceca content, suggesting factors crucial to the gut ecosystem, such as bile acids, SCFAs, humoral immune effectors, microbiota, neurochemical metabolites; all absent in their *in vitro* model, may influence this proposed pathway.

### Ceca Enterobacteriaceae Levels Associated With Intestinal IgA Levels

Given our observation of greater levels of Enterobacteriaceae in the PRO group, we hypothesized this had implications on host fecal shedding and mucosal responses. SISs from PRO birds contained the highest levels of anti-IutA and IroN IgA, associated with greater Enterobacteriaceae levels. IutA and IroN are iron siderophore receptors commonly found in extraintestinal pathogenic *E. coli*, which are commensals in the chicken intestine (Mellata, 2013). Thus, increases in these IgA may decrease risks for APEC infection by interfering with APEC translocation into the bloodstream at mucosal surfaces like the lung via connections between the gut-lung axis (Samuelson et al., 2015). Furthermore, why the RASV ablates the effect of probiotic-mediated increase in IgA and Enterobacteriaceae is unclear. Given the aforementioned decrease of SCFA-producing microbes in P+V birds, it is likely the ecological “restaurants” that break fiber down into accessible, simple-sugar substrates for Enterobacteriaceae are being altered (Conway and Cohen, 2015). Thus, vaccination with RASV may change these micro-niches within the GI tract, augmenting Enterobacteriaceae colonization, which is supported by the lower abundances of Enterobacteriaceae in the ceca and feces of vaccinated birds.

## Conclusion

**FIGURE 9:**
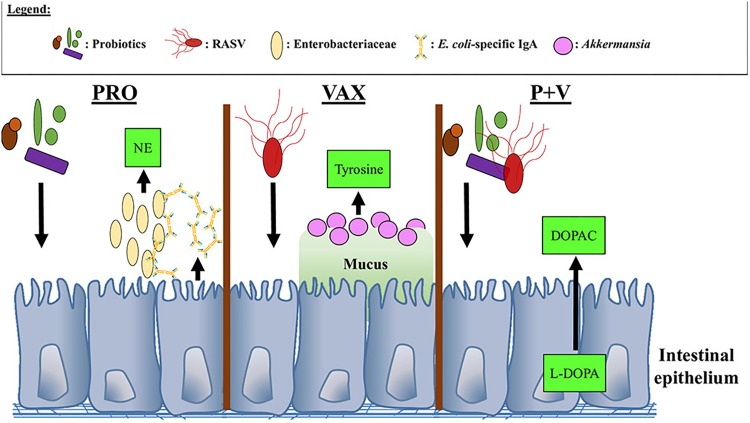
Proposed interactions between neurochemical metabolites, bacteria, and IgA in the chicken ceca. NE, Norepinephrine; DOPAC, 3,4-dihydroxyphenylacetic acid; L-DOPA, L-3,4-dihydroxyphenylalanine.

In conclusion, we show that treatment with probiotics and/or RASV results in unique catecholamine and microbiome profiles in the gut (summarized in Figure 9). Importantly, these changes appear to be related in certain circumstances and have implications on local humoral responses against particular pathogens. Additionally, we provide a provisional mechanism in which dopaminergic metabolism occurs in the chicken ceca. This study is the first to correlate neurochemical metabolites with microbiome data in the chicken model, which has important implications for disease susceptibility as well as behavior. Future studies will investigate how individual microbes within the probiotic mix as well as the RASV strain itself may contribute to these observed mechanisms, as well as using greater numbers of birds, which could be more representative of commercial poultry conditions.

## Data Availability Statement

The datasets generated for this study can be found in the NCBI Sequence Read Archive (SRA) repository with accession BioProject ID SUB5641933.

## Ethics Statement

The animal study was reviewed and approved by 1-16-8159-G.

## Author Contributions

MM conceived and designed the experiments. GR, KD, and MM performed the experiments. GR, KD, AS, ML, and MM analyzed the data. ML and MM contributed the reagents, materials, and analysis tools. GR and MM wrote the manuscript. KD, ML, and MM revised the manuscript. All authors read and approved the final version of the manuscript.

## Conflict of Interest

The authors declare that the research was conducted in the absence of any commercial or financial relationships that could be construed as a potential conflict of interest.

## Footnotes

1 https://github.com/ISUgenomics/MelhaMellata

## Supplementary Material

FIGURE S1 Fecal shedding of RASV χ9373 in chickens. Feces (*n* = 5 per group) were collected 1 week post-primary RASV immunization and plated on MacConkey agar for RASV enumeration. CON, no-treatment control. PRO, probiotics only. VAX, vaccine-only. P+V, vaccine and probiotics combination.

FIGURE S2 Impact of treatment on microbial alpha diversity in the chicken ceca (*n* = 10 per group) via **(A)** Faith’s PD (richness) and **(B)** evenness plots. Figures were generated via QIIME2 software. ^∗^*P* < 0.05. Yellow, PRO; blue, VAX; green, P+V.

FIGURE S3 Bacterial abundances of specific classes influenced by treatment group. 16S reads for Clostridia and Gammaproteobacteria were generated by QIIME2 software, and figures were developed on GraphPad. Yellow, PRO; blue, VAX; green, P+V. ^∗^*P* < 0.05; ^∗∗^*P* < 0.01; ^∗∗∗^*P* < 0.001.

FIGURE S4 Bacterial abundances by family influenced by treatment group. 16S reads for **(A)** Lachnospiraceae, **(B)** Peptostreptococcaceae, and **(C)** Mogibacteriaceae were generated by QIIME2 software, and figures were developed on GraphPad. Yellow, PRO; blue, VAX; green, P+V. ^∗^*P* < 0.05; ^∗∗^*P* < 0.01.

FIGURE S5 Bacterial genera influenced by treatment group. 16S reads for **(A)** *Enterococcus*, **(B)** *Weisella*, **(C)** *Anaerofustis*, **(D)** *Clostridium*, **(E)** *Coprabacillus*, and **(F)** *Cc-115* were generated by QIIME2 software, and figures were developed on GraphPad. Yellow, PRO; blue, VAX; green, P+V. ^∗^*P* < 0.05; ^∗∗∗^*P* < 0.001.

FIGURE S6 Gneiss heatmap used to determine taxonomic shifts in microbial balances specific to Probiotics (PRO), Live *Salmonella* (VAX), and Probiotics and Live *Salmonella* (P+V). Figure was generated via QIIME2 analysis.

FIGURE S7 Plots for comparing normality of tyrosine: *Akkermansia muciniphila* linear regression models generated by R software. **(A–D)** No log transformation of *A. muciniphila* 16S reads. **(E–H)** Log-transformed *A. muciniphila* 16S reads.

FIGURE S8 Plots for comparing normality of norepinephrine:Enterobacteriaceae linear regression models generated by R software. **(A–D)** No log transformation of Enterobacteriaceae 16S reads. **(E–H)** Log-transformed Enterobacteriaceae 16S reads.

TABLE S1 This table includes (i) original 16S abundance data, (ii) HPLC data, and (iii) log-transformed 16S abundance data for each individual bird, used for correlation analyses.

TABLE S2 This table includes output from Gneiss plugin from QIIME2 analysis describing balance shifts via treatments. PRO, probiotics only; VAX, vaccine only; P+V, vaccine and probiotics combination.
