# Supplementary figures and images for: Oral Treatments With Probiotics and Live Salmonella Vaccine Induce Unique Changes in Gut Neurochemicals and Microbiome in Chickens
Source: Front Microbiol. 2020 Jan 15;10:3064. doi: 10.3389/fmicb.2019.03064 (PMC6974472; doi:10.3389/fmicb.2019.03064)

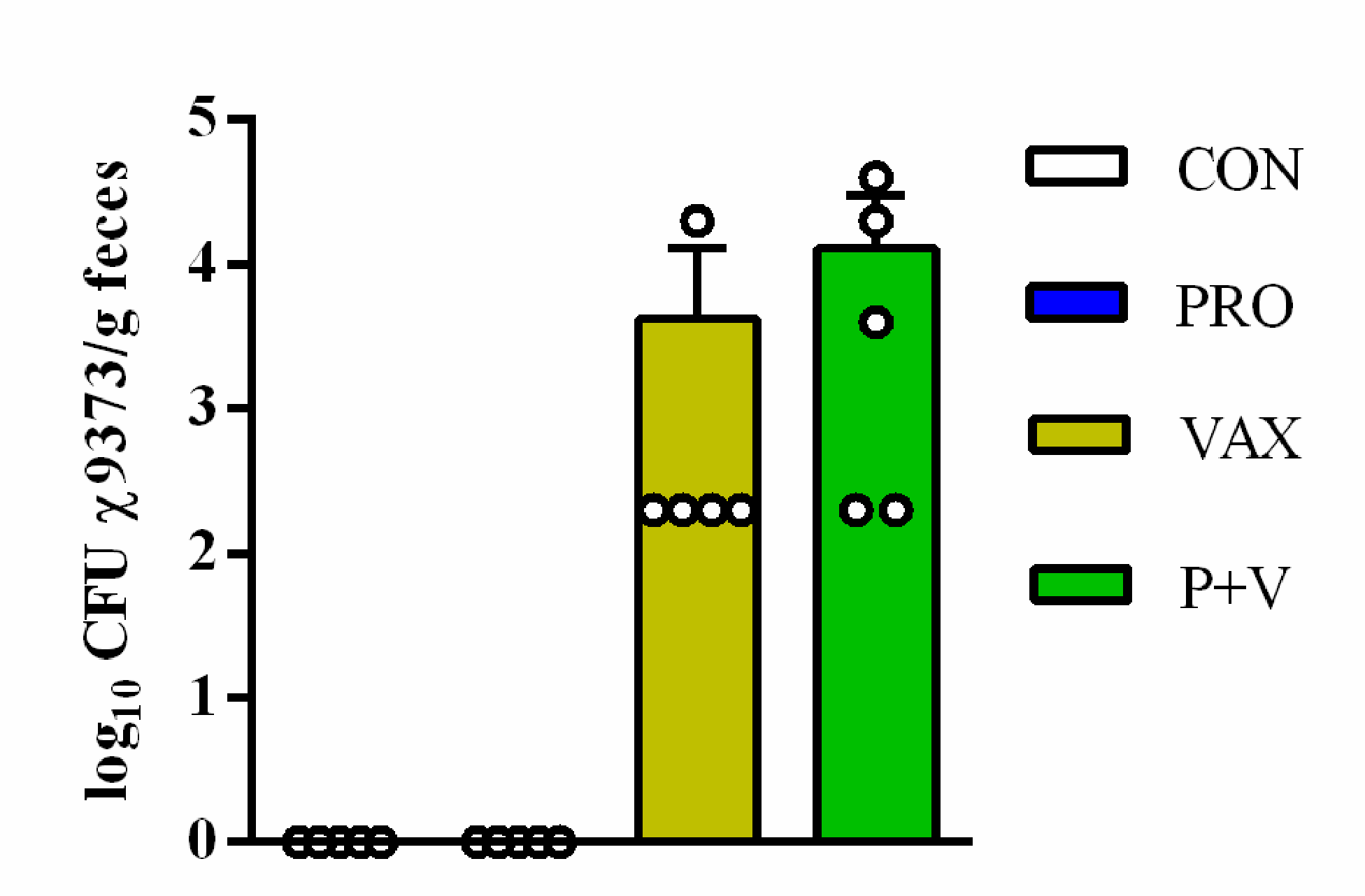

Supplement: FIGURE S1 — Fecal shedding of RASV χ9373 in chickens. Feces (n = 5 per group) were collected 1 week post-primary RASV immunization and plated on MacConkey agar for RASV enumeration. CON, no-treatment control. PRO, probiotics only. VAX, vaccine-only. P+V, vaccine and probiotics combination. [file Image_1.TIF]

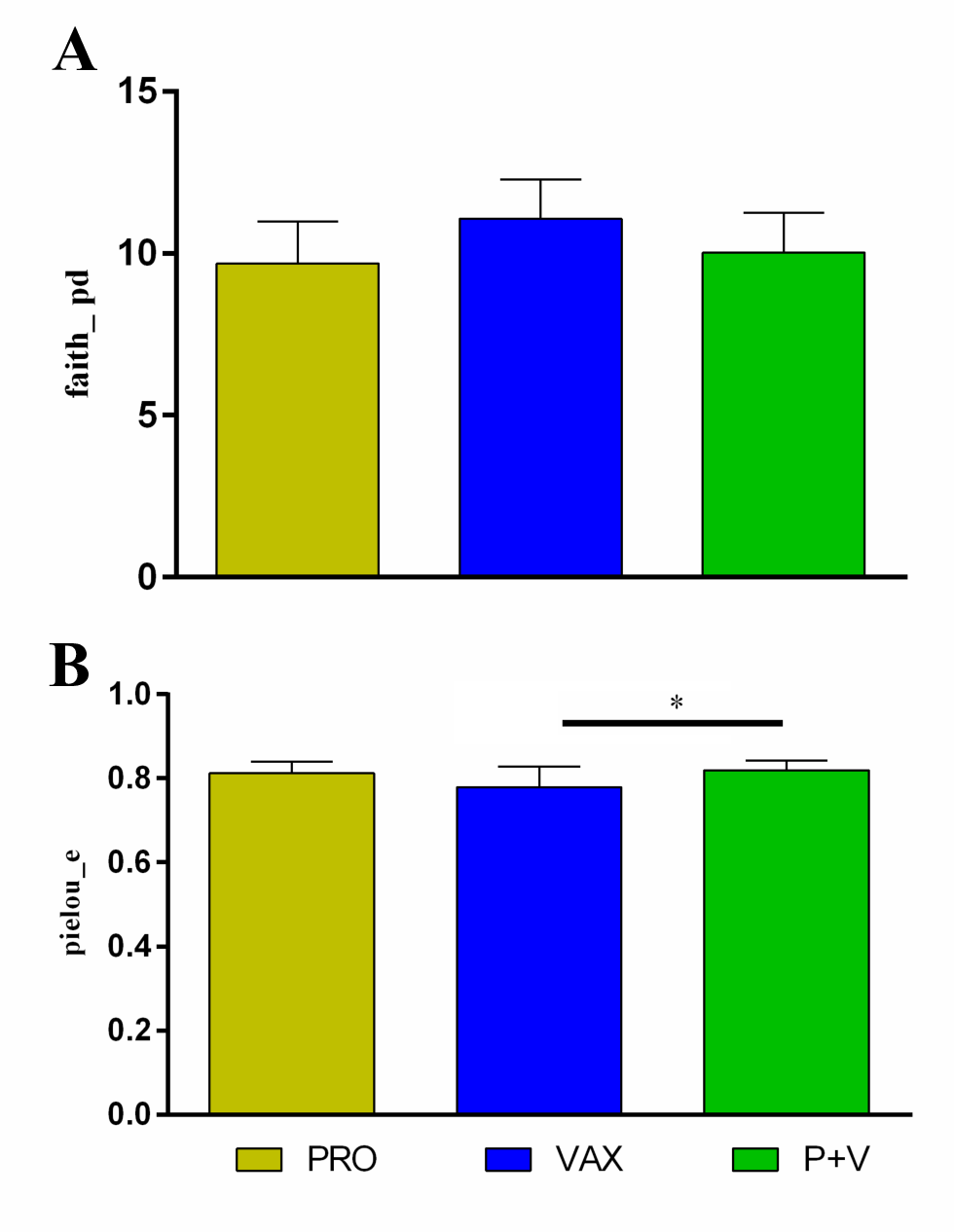

Supplement: FIGURE S2 — Impact of treatment on microbial alpha diversity in the chicken ceca (n = 10 per group) via (A) Faith’s PD (richness) and (B) evenness plots. Figures were generated via QIIME2 software. ∗P < 0.05. Yellow, PRO; blue, VAX; green, P+V. [file Image_2.tif]

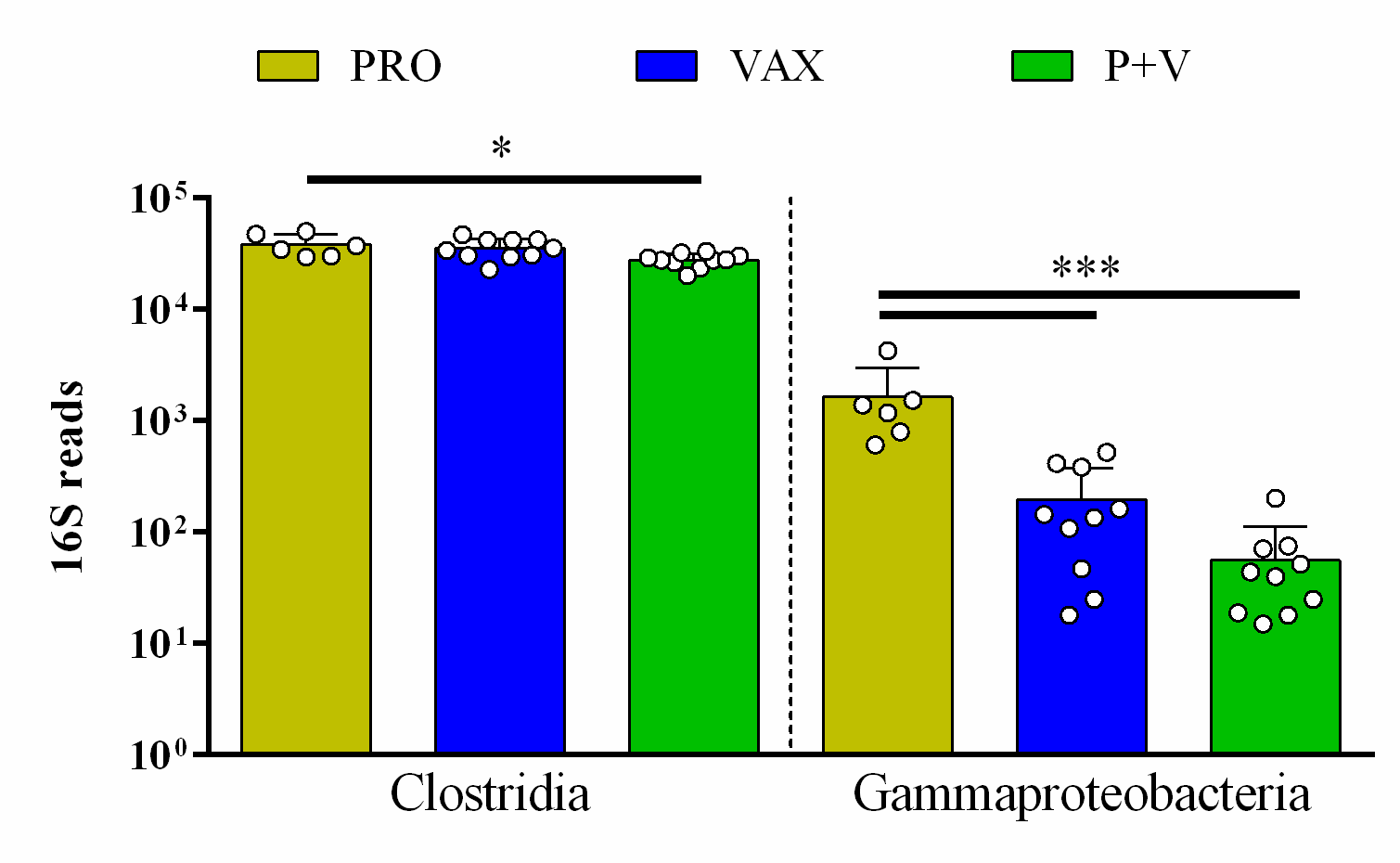

Supplement: FIGURE S3 — Bacterial abundances of specific classes influenced by treatment group. 16S reads for Clostridia and Gammaproteobacteria were generated by QIIME2 software, and figures were developed on GraphPad. Yellow, PRO; blue, VAX; green, P+V. ∗P < 0.05; ∗∗P < 0.01; ∗∗∗P < 0.001. [file Image_3.TIF]

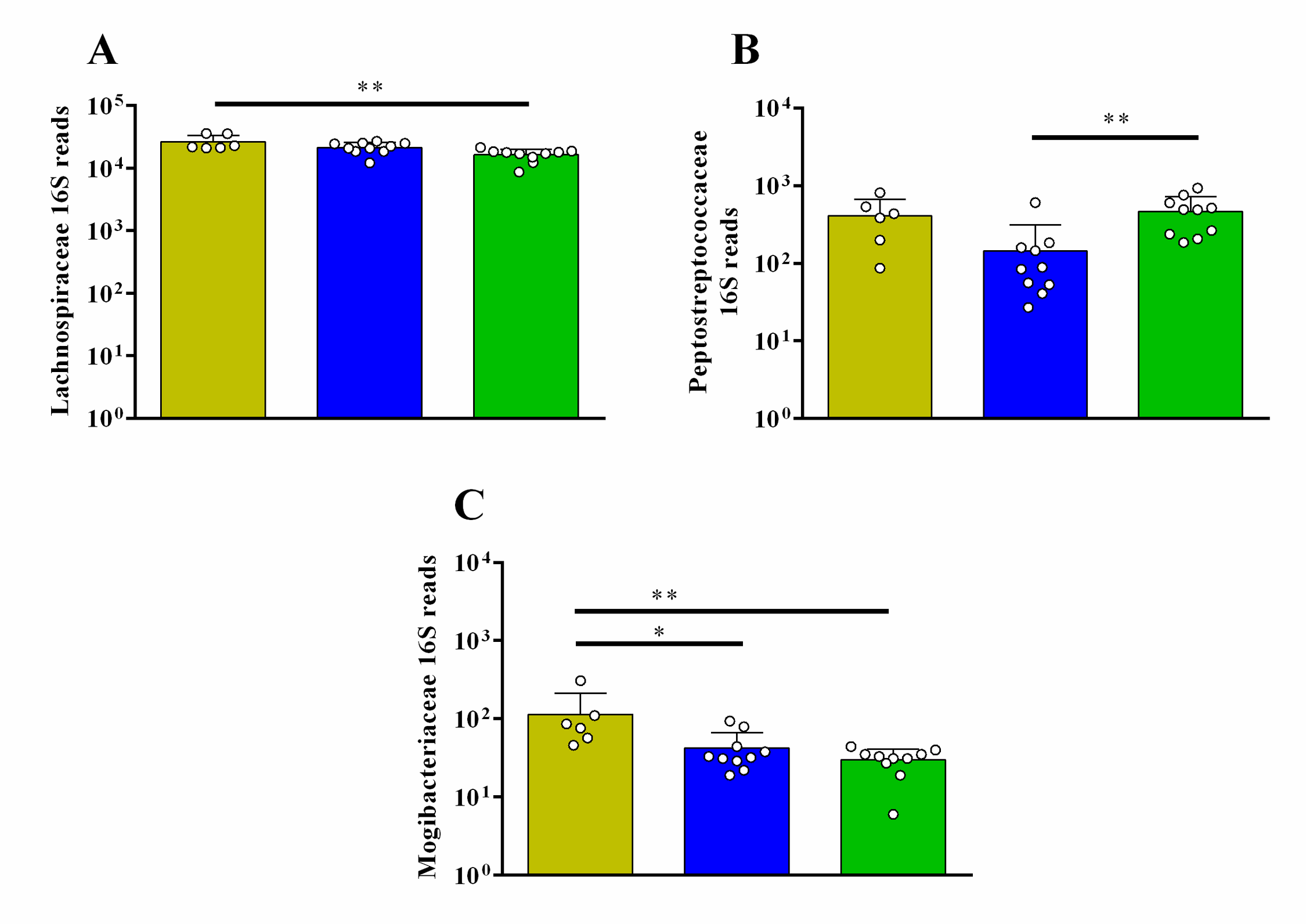

Supplement: FIGURE S4 — Bacterial abundances by family influenced by treatment group. 16S reads for (A) Lachnospiraceae, (B) Peptostreptococcaceae, and (C) Mogibacteriaceae were generated by QIIME2 software, and figures were developed on GraphPad. Yellow, PRO; blue, VAX; green, P+V. ∗P < 0.05; ∗∗P < 0.01. [file Image_4.TIF]

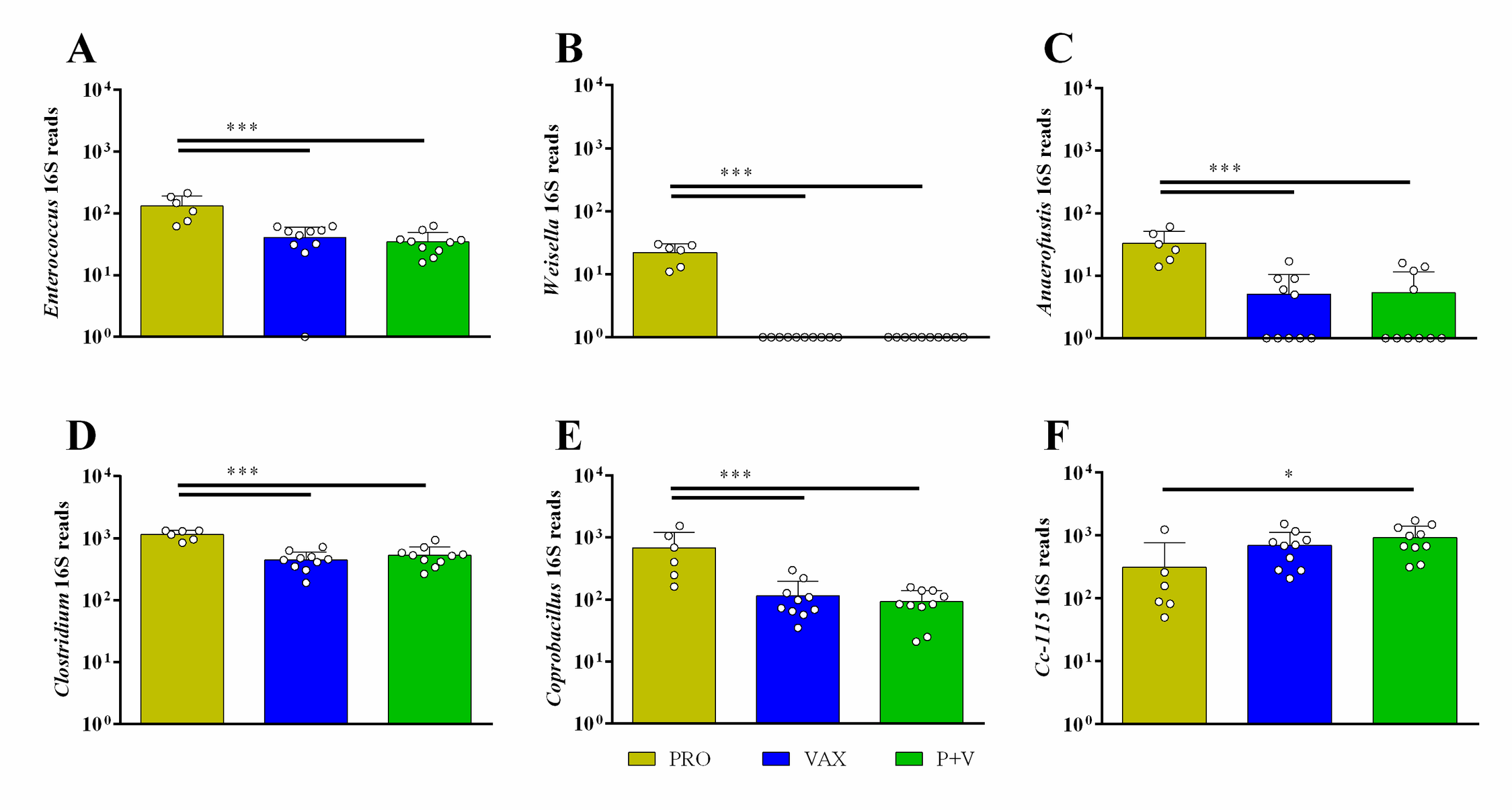

Supplement: FIGURE S5 — Bacterial genera influenced by treatment group. 16S reads for (A) Enterococcus, (B) Weisella, (C) Anaerofustis, (D) Clostridium, (E) Coprabacillus, and (F) Cc-115 were generated by QIIME2 software, and figures were developed on GraphPad. Yellow, PRO; blue, VAX; green, P+V. ∗P < 0.05; ∗∗∗P < 0.001. [file Image_5.TIF]

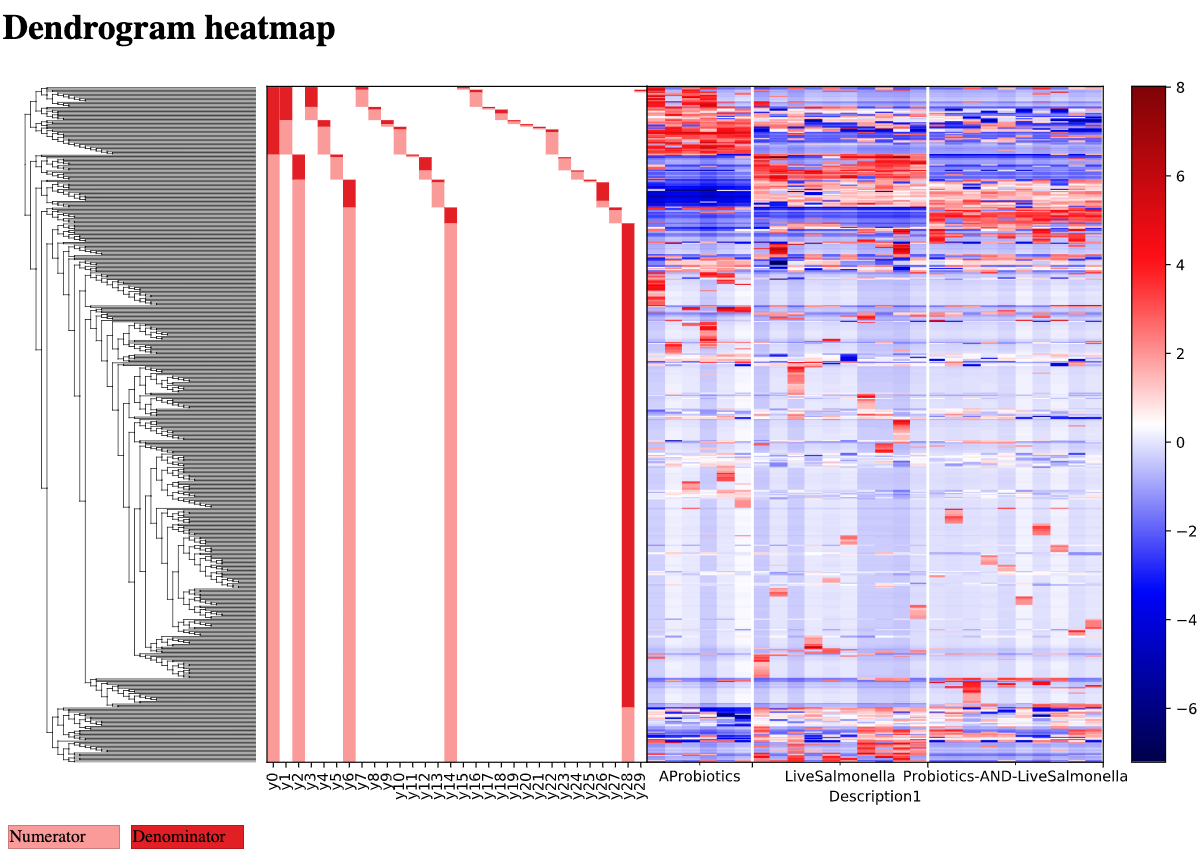

Supplement: FIGURE S6 — Gneiss heatmap used to determine taxonomic shifts in microbial balances specific to Probiotics (PRO), Live Salmonella (VAX), and Probiotics and Live Salmonella (P+V). Figure was generated via QIIME2 analysis. [file Image_6.TIF]

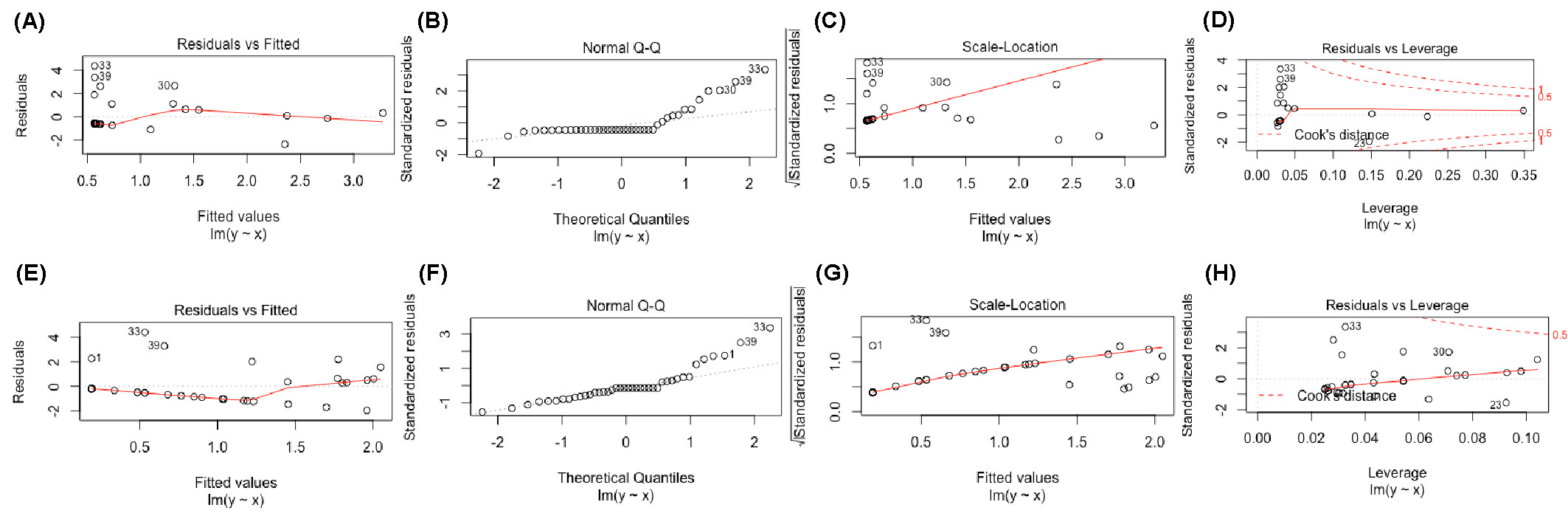

Supplement: FIGURE S7 — Plots for comparing normality of tyrosine: Akkermansia muciniphila linear regression models generated by R software. (A–D) No log transformation of A. muciniphila 16S reads. (E–H) Log-transformed A. muciniphila 16S reads. [file Image_7.jpg]

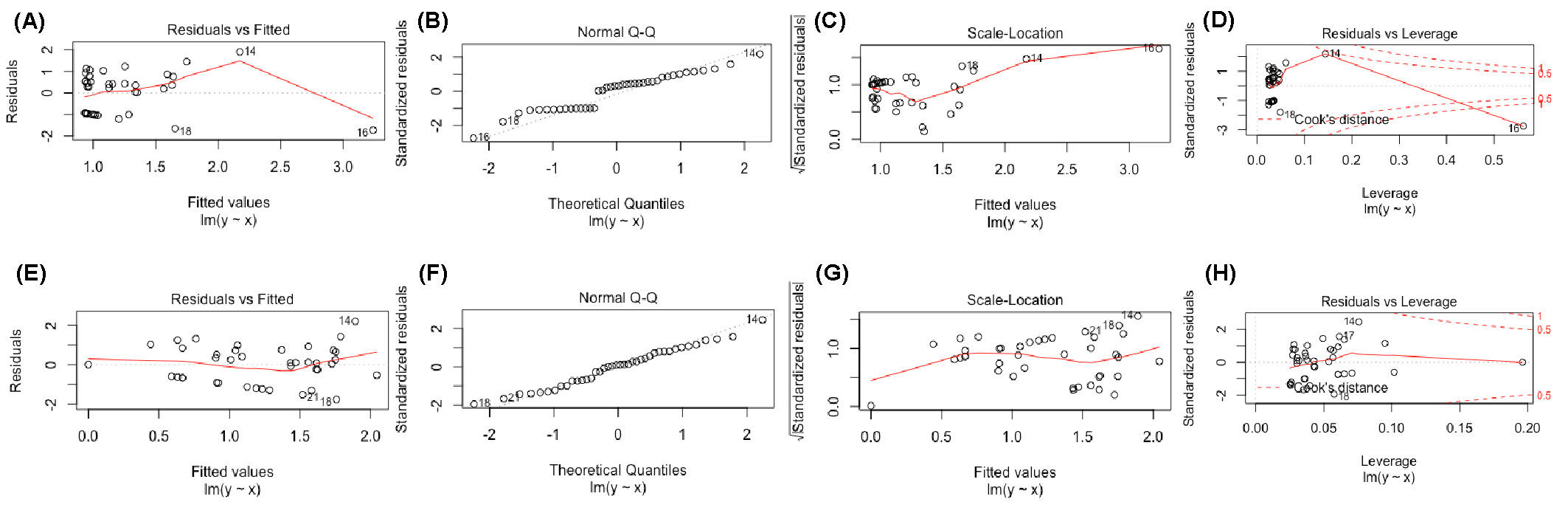

Supplement: FIGURE S8 — Plots for comparing normality of norepinephrine:Enterobacteriaceae linear regression models generated by R software. (A–D) No log transformation of Enterobacteriaceae 16S reads. (E–H) Log-transformed Enterobacteriaceae 16S reads. [file Image_8.jpg]
